# Decadal change in the association between the status of young mother’s Body Mass Index and anaemia with child low birth weight in India

**DOI:** 10.1186/s12884-022-04486-5

**Published:** 2022-02-22

**Authors:** Anuja Banerjee, Soumendu Sen, Junaid Khan, Manoranjan Pal, Premananda Bharati

**Affiliations:** 1 International Institute for Population Sciences, Govandi Station Road, Mumbai, 400088 India; 2 Indian Statistical Institute, 203 B.T. Road, Kolkata, 700108 India

**Keywords:** Decadal change, Body-mass index, Anaemia, Low birth weight, National Family Health Survey, India

## Abstract

**Background:**

The study aims to investigate the changes in the socio-economic and demographic status of young mothers of age 15–24 years and to examine the association between mothers’ nutrition, i.e., Body Mass Index (BMI) and anaemia with child low birth weight for almost two decades during 1998–2016 in India.

**Methods:**

National Family Health Survey (NFHS) round II and IV were used. The sample of this study included 3405 currently married young mothers from NFHS II and 44,742 from NFHS IV who gave birth at least one child in the last three years preceding the surveys. Logistic regression and Blinder-Oaxaca decomposition analysis have been used in this study to examine the corresponding association between the concerned variables.

**Results:**

The analysis showed that the prevalence of low birth weight (LBW) babies has decreased from 26.1 to 22.8 for the 15 to 19 age group and from 20.4 to 18.7 for the 20 to 24 age group over time. Young mothers with low BMI or severe anaemia have shown higher odds of having LBW babies. For instance, the odds of having a LBW child was 1.44 (*p*-value = 0.000; 95% CI: 1.05, 1.65) for mothers with low BMI and 1.55 (*p*-value = 0.000; 95% CI: 1.27, 1.90) with severe anaemia. Over the decade, the association of LBW babies with mothers’ nutrition has decreased. The odds of LBW with mothers with low BMI decreased from 1.63 (*p*-value = 0.004; 95% CI: 1.21, 2.21) to 1.41 (*p*-value = 0.000; 95% CI: 1.27, 1.55). Similarly, mothers with severe anaemia, the odds of LBW child decreased from 2.6 (*p*-value = 0.000; 95% CI: 1.75, 3.8) in 1998 to 1.3 (*p*-value = 0.024; 95% CI: 1.02, 1.65) in 2016.

**Conclusions:**

The maternal and child health improvement in India has been moderate over the decade. Still, a significant proportion of the women are suffering from poor health and young mothers are at more risk to deliver LBW babies. It is highly recommended to integrate maternal and child health programmes with the ongoing health policies to improve the situation while taking additional care of the young pregnant mother and their nutritional health.

## Introduction

Young mothers in India are disadvantaged in terms of their nutritional health and co-morbidities associated with their reproductive health [1, 2]. Biologically, about 50% of the adult body weight and 15% of the final adult height is attained during this adolescence, along with changes in body shape and composition. As a result, nutrition during this period is very important for overall growth. Poor nutrition starts before birth and generally continues into young and adult life and can span through the generations. It has been found that chronically malnourished women are more likely to remain undernourished during young and adulthood and when pregnant, are more likely to deliver low birth weight (LBW) babies [3]. An inadequate supply of nutrients during gestation is probably the single most important environmental factor affecting pregnancy outcomes. Previous studies suggest that the group of young girls, who conceive within two years of menarche and who get pregnant with low nutrient reserves and a short inter-pregnancy gap, tend to be at higher risk of having LBW babies which eventually become risky for mothers [4, 5].

Maternal undernutrition is prevalent in many regions, especially in south-central Asia, and in some countries, more than 10% of the women are shorter than 145 cm. Maternal undernutrition i.e., body-mass index (BMI) of less than 18·5 kg/m^2^—ranges from 10 to 19% in most of the countries from this region [6]. Similarly, a serious problem of maternal undernutrition is evident in most of the countries in Sub-Saharan Africa, South-Central and South-Eastern Asia, and in Yemen, where more than 20% of women have BMI which is less than 18·5 kg/m^2^.

In India, the prevalence of low BMI among women is 40% which is higher than in many developing nations. Studies revealed that the low BMI of mothers along with poor nutrition and maternal short stature are the potential risk factors for adverse birth outcomes and child’s LBW in particular [6]. Another study also found that the poor nutritional status among young women has important implications for physical work capacity and adverse reproductive outcomes and when those women enter with a large iron deficit and are subjected to the added demands for iron during pregnancy, it may be too late to address the problem [7].

Studies have confirmed that the nutritional status of Indian women is inadequate. For instance, at least 20% of women are thin (BMI < 18.5) and an equal proportion are overweight (BMI ≥ 23), 53% anaemic and 8% enter pregnancy as adolescents [8]. On the other hand, overnutrition has increased by 4 percentage points [9]. According to the National Family Health Survey (NFHS), the reported prevalence of anaemia among pregnant women ranges from 45 to 70% [8]. A study revealed that anaemia among mothers is highly associated with undernutrition and in turn resulting in LBW babies [10]. The young mother’s nutritional status is one of the most important determinants of child birth weight and BMI is a more pervasive factor across India [10]. Although the prevalence of young pregnancy has been reduced over time in India, still the number of pregnancies remain high. Mainly the young mothers of age groups 15 to 24 years are highly susceptible to LBW [11, 12]. The birth weight of a child is a reliable index of intrauterine growth retardation (IUGR) and a major factor determining a child’s survival, future physical growth, mental development and disease-free life [13]. LBW is found to be one of the major causes of high mortality and morbidity rate among infants [14]. Worldwide, more than 20 million births are counted as LBW every year, which accounts for 15 to 20% of the total live births [15]. In India, the prevalence of LBW infants has decreased from 20% in 2005–06 to 16% by 2015–16 [16]. The risk of perinatal mortality among LBW infants is several times higher than the infants with normal or higher birth weight [14].

Poor nutrition is one of the known facts behind the LBW of children in developing countries. Several other factors also affect the birth weight such as the age of the mother, economic status, birth order of the child, birth gap, smoking and drinking behaviour of the mother, antenatal care utilization [17]. LBW has several adverse consequences on an infant’s health. LBW babies are more likely to suffer from long term handicapping conditions, poor mental health, non-communicable diseases in later life [18, 19]. However, the main focus of this study is to examine the effect of nutritional health indicators of the mother i.e. BMI and anaemia on the child birth weight and how the association between these two factors and LBW has changed over nearly two decades.

## Data and methods

### Data source

The data sources that have been used in this study are the NFHS round II (1998–99) and the NFHS round IV (2015–16). Respective “Kids” files for the two different rounds were compiled for analysis. NFHS is a nationally representative Demographic and Health Survey (DHS) of India, which provides data on population, health, nutrition and other important indicators of population health. Data on BMI, anaemia level and birth weight were taken from both rounds. This study is carried out only for the currently married woman within the age of 15 to 24 years and who had delivered at least one child in the last three years preceding the date of the respective surveys. The following control variables have been considered from these two rounds viz. levels of highest education of the mother, wealth index, religion, caste, age group, place of delivery, birth order, type of delivery, place of residence, smoking chewing tobacco, alcohol consumption.

The data from two rounds were pooled to calculate the change in BMI and anaemia and the corresponding change in the odds of LBW babies with the same set of control variables. Although NFHS 2 has a smaller sample than NFHS 4, it does not lack the strength in terms of sample size (except for some socio-economic covariates where the sample size was less than 30 and the estimates were not provided for them) to provide national level estimates. Additionally, the distribution of birth weight variables from the two rounds have been studied and the skewness was examined. Dummies were created for each category of the independent variables and for each of the two time periods 1998–99 and 2015–16. Birth weight has been taken as a dependent variable which is divided into two categories i.e., LBW (< 2.5 kg) and normal or high birth weight (> = 2.5 kg) (WHO, 2014). According to WHO classification, BMI is categorized as low (< 18.5 kg/m^2^), normal (18.51 to 24.99 kg/m^2^) and high (> 25 kg/m^2^).

### Statistical analysis

Descriptive statistics, logistic regression and the Oaxaca decomposition method were used in this study. Given the fact that multivariate regression analysis techniques remain the most innovative and effective way to measure the associated change in the outcome variable due to unit change in the concerned outcome variable, we applied a multivariate regression analysis to examine the empirical association. To ensure the estimated beta coefficient/odds ratio gives an unbiased estimate of the effect of the concerned variable on the outcome variable, any statistical analysis should take care of the confounding effect in the system of equation. In this regard, an extended and in-depth information of the entities under study and an appropriate choice of the multivariate regression helps to achieve the goal. The literature review of this study supports the selection of covariates/confounders and like other Demographic Health Surveys (DHS), different rounds of NFHS for India provides detailed information on socio-economic and demographic characteristics of the respondents/children. Taking care of the nature of the dependent variable, we chose a multivariate logistic regression analysis with the underlying assumptions of a logistic regression in this study. Essentially, the dependent variable is a dichotomous variable, the observations are independent, there are no multicollinearity among the explanatory variables, no extreme outliers are present in the dataset. Hence, this study also assumed a linear relationship between the logit of the response variable and the sum of the explanatory variables.

Logistic regression was run to regress the birth weight with the given set of independent variables. The pooled regression analysis was run with the prior identification of all the categories of BMI and anaemia which were eventually the time specified dummies. To show the association between birth weight with BMI, anaemia and socio-demographic variables, a binary logistic regression model was fitted. The model used is-

$${\mathrm{Y}}_{\mathrm{i}}=\mathrm{ ln }[\mathrm{P}/ (1-\mathrm{P})] = {\upbeta }_{0}+ {\upbeta }_{1}{\mathrm{X}}_{1}+ {\upbeta }_{2}{\mathrm{X}}_{2}\dots . + {\upbeta }_{\mathrm{n}}{\mathrm{X}}_{\mathrm{n}},$$

where Yi is the log odds ratio of P, the probability of LBW (1 if the baby has LBW otherwise 0), *β*_*0 *_represents the intercept term of the model, *β*_*i*_(where i = 1,2,3, …, n) is the coefficient of i-th variable X_i_ (where i = 1,2,3…n). The independent variables are as follows: age (15 to 19, 20 to 24), location (urban, rural), caste (Scheduled castes [SC], Scheduled tribes [ST], Other backward classes [OBC], general), religion (Hindu, Muslim, Others), education (no education, primary, secondary, higher), wealth index (poor, middle, rich), time (NFHS-II, NFHS-IV), birth order (1, 2, 3 +), place of delivery (institutional, non-institutional), delivery by caesarian section (yes, no), smoking (yes, no), drinking alcohol (yes, no) and chewing tobacco (yes, no).

The decomposition method outlined in this part of the study, known as the Oaxaca decomposition, which is also known as Blinder-Oaxaca decomposition [20, 21], explains the gap in the means of an outcome variable between two groups. The gap was decomposed into two parts – one part explains the effect of the differences in the means of inputs between two groups and the other part is due to the differential effects of the two groups. The decomposition of birth weight was studied for different BMI and anaemia levels of the mothers.

## Results

### Change in basic characteristics of young mothers

Table 1 shows the background characteristics of young mothers in India in two different time periods. The proportion of mothers in the 15 to 19 age group with no education decreased over the years from 26.4% to 15.2% whereas the proportion share remained almost the same for the 20–24 age group of mothers. It is also observed that the percentage of mothers belonging to the poor category has increased over the years substantially to 53.5% from 37.6% in the 15 to19 years age group and the percentage reached 41.2% from 36.4% for the 20 to24 age group of mothers. In a similar direction, the proportion of young mothers from the rich wealth quintile has shown a decline over time for both age groups. On the contrary, though the percentage of young mothers in the urban areas declined, the percentage in rural areas has increased. The proportion of mothers with normal BMI and not anaemic has increased over time. Furthermore, institutional delivery has increased from 1998 to 2016 but delivery by c-section among young mothers remained almost the same.

**Table 1:** Background characteristics of young mothers age 15–24, India, NFHS, 1998–99 & 2015–16

	Young mothers (15–19)				Young mothers (20–24)			
	**1998–99**		**2015–16**		**1998–99**		**2015–16**	
	**%**	**N**	**%**	**N**	**%**	**N**	**%**	**N**
**Highest education**								
No education	26.36	153	15.18	726	16.17	432	16.51	7269
Primary	26.45	155	12.07	521	16.92	464	13.33	5580
Secondary	44.9	278	71	2915	48.8	1395	60.21	23,972
Higher	2.29	13	1.75	71	18.1	515	9.95	3688
**Wealth index**								
Poor	37.65	226	53.51	2364	36.41	983	41.19	17,969
Middle	19.19	113	25.29	1009	20.79	596	24.28	9517
Rich	43.16	260	21.2	860	42.8	1227	34.53	13,023
**Religion**								
Hindu	80.18	450	81.52	3327	79.99	2106	83.45	32,805
Muslim	16.2	90	13.87	530	13.47	353	12.16	4456
Others	3.62	59	4.61	376	6.54	347	4.39	3248
**Caste**								
SC	18.7	113	25.92	962	17.4	460	22.99	8484
ST	5.8	74	13.56	890	5.38	294	11.3	6897
OBC	37.37	198	40.84	1703	38.43	935	46.13	18,055
GENERAL	38.13	214	19.68	678	38.79	1117	19.57	7073
**Type of residence**								
Urban	29.01	208	19.86	759	41.37	1337	26.26	9155
Rural	70.99	391	80.14	3474	58.63	1469	73.84	31,354
**BMI**								
High	2.7	21	6.32	202	6.63	187	9.96	3599
Normal	51.43	330	58.89	2530	56.62	1686	60.83	25,346
Low	45.87	248	34.79	1501	36.75	933	29.2	11,564
**Anaemia**								
Not Anaemic	27.9	183	39.45	1653	35.55	1049	42.21	17,094
Mild	27.4	150	45.92	1907	24.61	695	42.54	17,208
Moderate	40.93	244	13.34	627	36.08	961	14.43	5875
Severe	3.77	22	1.29	46	3.75	101	0.82	331
**Birth order**								
1 order	81.79	506	88.59	3764	56.47	1634	58.9	24,246
2 order	15.84	82	10.81	444	31.07	850	32.78	12,851
3 + order	2.37	11	0.6	25	12.47	319	8.32	3412
**Place of delivery**								
Non-institutional	9.35	54	6.29	272	7.45	234	6.55	2833
Institutional	90.64	545	93.71	3961	92.55	2572	93.45	37,676
**C-section delivery**								
No	80.59	455	81.16	3616	79.35	2141	79.75	33,966
Yes	19.41	112	18.84	617	20.65	516	20.25	6543
**India**	**26.08**	**599**	**20.4**	**4233**	**22.76**	**2806**	**18.67**	**40,509**

### Change in mean birth weight by BMI

It has been found that with an increase in BMI levels, the mean birth weight of the child has also increased. It is lowest for a woman having low BMI for both the age group of mothers (2.67 kg for 15–19 age group and 2.7 kgs for 20–24 age group). But over the years, we found that the changes in mean birth weight are not conspicuous in any of the groups, which is expected. Similarly, it has been found that the anaemia level of mothers and mean birth weight of their child followed an inverse relationship. Mothers with severe anaemia showed the lowest mean birth weight for both age groups. For instance, it was 2.4 kgs for both age-group in 1998–99 and 2.6 kgs and 2.7 kgs in 15–19 and 20–24 age group respectively in 2015–16.

Table 2 presents the prevalence of LBW of young mothers in India at two different periods. The prevalence of LBW has decreased from 26.08% in 1998–99 to 20.4% in 2015–16 for mothers with age 15 to 19 years. Similarly, for the 20 to 24 years age group, the LBW prevalence has decreased from 22.76% to 18.67% for the same time period. The prevalence of LBW has shown a reduction for all socio-economic covariates and all age-group over the two periods but with some exceptions. For the 15 to 19 age group, the prevalence has increased for mothers with no anaemic condition, belonged to schedule tribe, non-institutional and caesarian delivery.

**Table 2:** Prevalence of LBW among young mothers by socio-economic covariates in India, 1998- 2016

	Young mothers (15–19)						Young mothers (20–24)					
	1998–99	N	*p*-value	2015–16	N	*p*-value	1998–99	N	*p*-value	2015–16	N	*p*-value
**BMI**												
High	NA	NA	0.081	5.8	202	0.000	11.4	186	0.000	15.6	3599	0.000
Normal	26.9	327		20.2	2530		21.1	1663		17.8	25,346	
Low	26.3	245		23.4	1501		27.3	915		21.6	11,546	
**Anaemia**												
Not anaemic	17.4	182	0.001	20.3	1653	0.227	19.5	1038	0.000	18.4	17,094	0.000
Mild	23.6	147		19.6	1907		20.8	680		18.1	17,208	
Moderate	32.4	242		23.5	627		25.4	946		20.9	5875	
Severe	NA	NA		20.7	46		40.7	100		23.3	331	
**Place of residence**												
Urban	24.7	204	0.004	16.0	759	0.079	21.0	1321	0.000	17.8	9155	0.000
Rural	26.7	389		21.5	2474		24.0	1443		19.0	31,354	
**Caste**												
SC	31.6	113	0.052	21.0	962	0.334	26.3	452	0.189	19.0	8484	0.000
ST	16.5	72		25.5	890		25.8	282		21.5	6897	
OBC	22.1	196		20.9	1703		22.2	929		18.3	18,055	
General	28.7	212		15.0	678		21.3	1101		17.6	7073	
**Religion**												
Hindu	25.8	445	0.706	21.0	3327	0.001	23.7	2082	0.008	18.9	32,805	0.000
Muslim	24.0	90		16.2	530		18.0	347		17.7	4456	
Others	42.1	58		21.9	376		21.5	335		17.3	3248	
**Education**												
No education	24.8	152	0.311	22.0	726	0.008	32.3	424	0.000	21.5	7269	0.000
Primary	30.2	153		23.6	521		22.9	455		19.7	5580	
Secondary	25.4	275		19.6	2915		22.1	1373		18.1	23,972	
Higher	NA	NA		19.2	71		16.0	512		16.1	3688	
**Wealth quintile**												
Poor	26.3	223	0.151	22.4	2364	0.016	22.6	965	0.472	20.0	17,969	0.000
Middle	31.4	111		18.1	1009		22.3	585		18.3	9517	
Rich	23.6	259		18.0	860		23.1	1214		17.4	13,023	
**Birth order**												
1 order	25.2	500	0.305	20.2	3764	0.773	21.6	1603	0.005	19.3	24,246	0.002
2 order	28.0	82		22.0	444		23.1	843		17.8	12,851	
3 + order	NA	NA		NA	NA		27.3	318		18.1	3412	
**Place of delivery**												
Non-institutional	17.2	53	0.575	25.4	268	0.186	32.9	222	0.026	20.7	2759	0.021
Institutional	27.1	539		20.1	3961		22.0	2535		18.5	37,676	
**Delivery by caesarian section**												
No	27.8	450	0.078	21.1	3616	0.263	22.4	2107	0.717	18.7	33,966	0.056
Yes	16.4	111		17.6	617		21.9	509		18.4	6543	
**India**	**26.1**	**599**		**20.4**	**4233**		**22.8**	**2764**		**18.7**	**40,509**	

Estimates are not available (NA) for sample size of less than 30

### Association of BMI, anaemia and other determinants with low birth weight

Table 3 presents the odds ratio of LBW with the nutritional status of young mothers and other socio-economic covariates. The odds of having LBW babies are 1.15 (95% CI: 1.3, 1.58) and 1.44 (95% CI: 1.05, 1.65) for mothers with normal and low BMI respectively. Similarly, mothers with severe anaemia and moderate anaemia have 55% and 14% more likely to have LBW babies than no anaemic mothers respectively. Mothers with ages 20 to 24 years who belong to scheduled tribe and OBC categories, secondary or higher educated are less likely to have LBW children. However, no significant association has been found with the rest of the wealth tertile (middle & rich) and LBW children compared to the poor wealth tertile. The odds of having LBW babies is 1.13 (95% CI: 1.06, 1.2) for mothers who delivered by caesarian section.

**Table 3:** Association of BMI, anaemia and other determining variables with LBW, India 1998–2016

Determinants	Odds Ratio	95% CI
**BMI** (High BMI®)		
Normal BMI	1.15**	(1.3,1.58)
Low BMI	1.44***	(1.05,1.65)
**Anaemia level** (Not anaemic ®)		
Mild	1.01	(0.96,1.07)
Moderate	1.14***	(1.06,1.22)
Severe	1.55***	(1.27,1.9)
**Age** (15–19®)		
20–24	0.93*	(0.86,0.99)
**Location** (Urban®)		
Rural	0.97	(0.91,1.03)
**Caste** (SC®)		
ST	0.90**	(0.83,0.97)
OBC	0.91**	(0.86,0.97)
General	0.95	(0.88,1.03)
**Religion** (Hindu®)		
Muslim	0.88**	(0.82,0.96)
Others	0.66***	(0.6,0.73)
**Education** (No education®)		
Primary	0.94	(0.87,1.02)
Secondary	0.79***	(0.74,0.84)
Higher	0.66***	(0.59,0.73)
**Wealth index** (Poor®)		
Middle	0.96	(0.91,1.02)
Rich	0.99	(0.93,1.06)
**Time** (NFHS 2®)		
NFHS4	0.8***	(0.73,0.88)
**Birth order** (1 order®)		
2 order	0.89***	(0.85,0.94)
3 order +	0.92*	(0.84,0.99)
**Place of delivery** (Non-institutional®)		
Institutional	0.87**	(0.79,0.95)
**Delivery by caesarian section** (No ®)		
Yes	1.13***	(1.06,1.2)
**Smoking** (No®)		
Yes	1.06	(0.58,1.93)
**Drinking alcohol** (No®)		
Yes	0.97	(0.79,1.2)
**Chewing tobacco** (No®)		
Yes	0.88	(0.69,1.13)

^*^p =  < 0.1, **p < .05, ***p < .01

### Change in association between BMI, anemia and low birth weight over the decade

Table 4 shows the change in the association between BMI, anaemia and birth weight over almost two decades of the study period. The association of BMI on birth weight has changed over the years. We can deduce that the likelihood of having LBW babies for a woman with low and normal BMI has decreased over the years when controlled for all other socio-economic demographic characteristics of the mothers. During 1998–99, the odds of having LBW babies were 1.63 and 1.49 for a woman having low and normal BMI respectively and during 2015–16 the odds were 1.41 and 1.12 respectively. It indicates that the chances of having LBW babies for a woman with a low BMI was 63% more likely and for a woman, with normal BMI it was 49% more likely. But during NFHS-2015–16, the chances or likelihood have shown a reduction. LBW babies are now 41% and 12% more likely to occur to a woman with a low and normal BMI.

**Table 4:** Change in the association of LBW Babies with the Status of BMI and Anemia of Mothers, India 1998–2016

Variables	OR	95% CI	
**Nutritional characteristics**			
**Anaemia level**			
Not anaemic in NFHS 2 & NFHS 4®			
Severe in NFHS 2	2.6***		(1.75, 3.8)
Severe in NFHS 4	1.30**		(1.02, 1.65)
Moderate in NFHS 2	1.39***		(1.15, 1.69)
Moderate in NFHS 4	1.11**		(1.03, 1.18)
Mild in NFHS 2	1.08		(0.87, 1.34)
Mild in NFHS 4	1.03		(0.98, 1.09)
**BMI**			
High BMI in NFHS 2 & in NFHS 4®			
Low BMI in NFHS 2	1.63**		(1.21,2.21)
Low BMI in NFHS 4	1.41***		(1.27,1.55)
Normal BMI in NFHS 2	1.49**		(1.11,1.98)
Normal BMI in NFHS 4	1.12**		(1.02,1.24)

^*^p =  < 0.1, **p < .05, ***p < .01. *N* = 48,099, All other variables are controlled

For anaemia, the association with birth weight has decreased over the years. During 1998–99, the chances of having LBW babies was 2.6 times likely to occur to a woman with severe anaemia and 39% more likely to occur to a woman with moderate anaemia. Whereas during 2015–16, we can observe that the association has decreased over time. Women with severe and moderate anaemia are 30% and 11% more likely to have LBW babies. The likelihood of LBW babies among women with anaemia seemed to have decreased during 2015–16.

### Blinder Oaxaca decomposition of birth weight with respect to BMI and anemia

Table 5 presents the results from the Oaxaca decomposition of birth weight in India. The principal idea behind decomposition is to explain the distribution of the outcome variable through a set of factors that vary systematically with the socioeconomic status. This technique decomposes the gap between the average outcome variable in two population groups into two components. One component is due to the differences in the magnitudes of the determinants of the outcome between populations (the explained or endowment component) and the second component is the unexplained component that arises due to group differences in the effects of this determinant. The difference in co-efficient of the outcome variable, here the birth weight, when observed with respect to BMI, is 0.11 units. Blinder Oaxaca decomposition helps to decompose the difference in birth weight between two groups of women in terms of their BMI status (low BMI or not low BMI) and the difference was further decomposed into explained and unexplained parts by that particular explanatory variable (BMI status of the woman). Prediction 1 gives the average response of birth weight for those women who do not fall in the low BMI category whereas prediction 2 is the average response of the counter category. The basic difference between the two groups was found to be 0.11 units. Further decomposition of this difference on the account of mother’s BMI status showed that only 18.18% (co-efficient 0.02) of the total difference can be explained by the BMI status of women, whereas the rest of the part remained unexplained and can be explained by other factors affecting birth weight which was not the aim of the study.

**Table 5:** Blinder Oaxaca Decomposition of Birth Weight with respect to BMI and Anemia, India 1998–2016

**BMI**		
Birth weight	Coefficient	% explained
Prediction_1 (not low BMI)	2.81***	-
Prediction_2 (low BMI)	2.70***	-
Difference	0.11***	-
Decomposition		-
Explained	0.02***	18.18
Unexplained	0.09***	81.82
**Anaemia**		
Birth weight	Coefficient	% explained
Prediction_1 (no severe anaemia)	2.78***	-
Prediction_2 (severe anaemia)	2.63***	-
Difference	0.14***	-
Decomposition		-
Explained	0.05***	35.71
Unexplained	0.09**	64.29

^*^p =  < 0.1, **p < .05, ***p < .01. *N* = 49,405

However, we also observed that the difference in mean birth weight with respect to anaemia is 0.14 units. Out of which only 35.71% (coefficient = 0.05) of the difference can be explained by anaemia and the rest of it remained unexplained due to other factors affecting Birth Weight.

## Discussion

This paper examined the association of young mothers’ nutritional status with childbirth weight. In this study, those mothers were considered who became pregnant quite early between 15 to 24 years of their age and were exposed to deliver LBW children. The major findings of this study are as follows.

First, the prevalence of low BMI and severe anaemic mothers have decreased over the decades. Second, the birth weight of the infants is directly related to BMI but inversely related to the anaemia level of the mothers. Third, over the decade, the prevalence of LBW babies has decreased and a higher reduction has taken place for mothers within the age of 15 to 19 years. Fourth, the odds of having LBW babies was significantly higher for mothers with low BMI, moderate and severe anaemia. However, mild anaemic mothers do not show any significant association. Moreover, the association between BMI, anaemia and child birth weight has decreased over the decade. Fifth, BMI can explain about one-fifth of the difference in child birth weight between mothers with low and normal BMI whereas more than one-third of the difference can be explained by anaemia between mothers with severe and non-severe anaemia.

Some possible explanations of these findings have been provided. Women with abnormal BMI are at risk of LBW outcomes [22]. The decrease of low BMI and severe anaemic women over the decade can be explained by the increase in educational attainment and standard of living which in turn improved the dietary intake of the young mothers. Education is one of the most important and significant factors which plays a substantial role to improve the nutritional status of women [23, 24]. Along with educational attainment, a household’s wealth status shows high association with BMI and anaemia [25, 26]. Our study results show a similar pattern as the studies conducted in other developing countries. A study in a Sudan hospital found that lack of antenatal care and anaemic mothers were the significant determinants of LBW [27]. Another study in Nepal revealed that normal BMI was the protective factor against the child's LBW [28]. In Ethiopia, a study by Betew et al. in 2014 analyzed the determinants of LBW by using the same Demographic and Health Survey data. They concluded that the children born from lower education, low income, anaemic, low BMI and teenage mothers had a higher risk of delivering children with LBW [29]. Though the pattern of the association between mother’s BMI and anaemia with child's LBW remained the same over time but the intensity of association has reduced. There also exists regional variation in BMI and anaemia status of women. For instance, women residing in villages are more likely to have low BMI and suffer from anaemia. In this study, it is found that the prevalence of LBW babies has decreased over the decade. Increased educational status through programs in TV and newspaper articles have a significant role in reducing LBW babies [30]. Another study has found that regular antenatal check-ups and balanced diet intake during the antenatal period have a larger impact on the reduction of LBW infants [31].

Since the implementation of the National Health Mission (NHM) in 2005, the improvement in maternal and child health has been accelerated [32]. The effects of the introduction of such programs have major impacts on maternal and child health. Though public health interventions are making efforts to improve maternal health by reducing maternal mortality but still a large proportion of the women are suffering from poor health and young mothers are at more risk. BMI status of the mother is a major predictor of foetal growth and maternal poor health increases the risk of stillbirth and neonatal death [33]. To combat the situation of low BMI and anaemia among young mothers and pregnancy at a very early stage of their life, India needs to employ multidimensional intervention programmes based on identified individual, household and community-level socio-demographic and economic risk factors that affect maternal nutritional status. Schemes like National Rural Health Mission, Reproductive Maternal Newborn and Child Health + Adolescent (RMNCH + A) in India are working on maternal and child nutrition care. Mother and Child Protection cards of the Ministry of Health and Family Welfare and Ministry of Woman and Child Development are being used by all states as a tool for monitoring and improving the quality of maternal and child health and nutrition interventions. In India, there are some schemes and programs which have the following interventions to promote women’s nutrition: counselling on adequate nutrition during antenatal and postnatal contacts; and provision of iron-folic acid supplements to pregnant women. India may consider replacing iron-folic acid with multiple-micronutrient supplements to all pregnant women, provision of calcium supplementation to those at risk of low intake and provision of balanced energy–protein supplementation to pregnant women as needed, as recommended in the second Lancet series on maternal and child nutrition [34]. Regular systematic monitoring and surveillance of the social trajectory of the nutritional status of young women are essential to develop an appropriate strategy to reduce the burden of LBW outcomes in India.

This study has the following limitation. As the data was taken from a cross-sectional survey which provides only a snapshot of any current situation, the BMI and anaemia level of the mother was captured at the time of interview and not during their pregnancy period.

## Data Availability

The study utilizes secondary data sources which is publicly available through URL: https://www.dhsprogram.com/data/available-datasets.cfm.

## Abbreviations

LBW: Low birth weight
BMI: Body-mass index
NFHS: National Family Health Survey
DHS: Demographic Health Survey
